# Machine Learning–Based Prediction of Culture-Confirmed Neonatal Sepsis in a Tertiary Neonatal Intensive Care Unit: Retrospective Cohort Study

**DOI:** 10.2196/88732

**Published:** 2026-09-14

**Authors:** Eman Badran, Oraib Al-Smadi, Loiy T Algazo, Alaa T Al Ghazo, Arwa Al Anber, Areej Sharaqa, Lena Abu-Argoub, Shatha Al Jaberi, Abdulrahman Alhanbali, Taimein Yacoub, Shahd Rihan, Hala Al-Jaberi

**Affiliations:** 1 Department of Pediatrics, Neonatal Division, School of Medicine, University of Jordan, Queen Rania Street, Amman, 11942, Jordan, 962 795608288, 962 6 5300431; 2 Department of Neonatology, Princess Rahmah Teaching Hospital, Ministry of Health, Irbid, Jordan; 3 Department of Mechatronics Engineering, Faculty of Engineering, Hashemite University, Zarqa, Jordan; 4 Department of Pharmacology Public Health and Clinical Skills, Faculty of Medicine, Hashemite University, Zarqa, Jordan; 5 Department of Pediatrics, The University of Texas Medical Branch at Galveston, Galveston, TX, United States

**Keywords:** machine learning models, Extreme Gradient Boosting, XGBoost, neural networks, decision trees, electronic health record, EHR, neonatal sepsis, clinical decision support tools

## Abstract

**Background:**

Neonatal sepsis remains a major cause of neonatal morbidity and mortality in low- and middle-income countries (LMICs). Early diagnosis is challenging because of nonspecific clinical manifestations and delays in laboratory confirmation. Machine learning (ML) approaches using structured electronic health record (EHR) data may improve early risk stratification in neonatal intensive care units (NICUs).

**Objective:**

This study aimed to evaluate ML models for predicting culture-confirmed neonatal sepsis among neonates admitted to a tertiary NICU in Jordan, with the objective of addressing diagnostic gaps in resource-limited settings. Specifically, we aimed to identify key predictors through feature importance analysis, evaluate model performance with class-imbalanced data, and propose strategies to improve interpretability and generalizability in LMICs.

**Methods:**

A retrospective cohort study was conducted using structured EHRs of 3274 neonates admitted to a tertiary NICU in Jordan between 2018 and 2024. Neonates who underwent blood culture testing were included. The dataset was divided into training (n=2619, 80%) and testing (n=655, 20%) subsets using stratified sampling. Three ML models—Extreme Gradient Boosting (XGBoost), decision trees, and neural networks—were trained using clinical, laboratory, and demographic variables. Class imbalance was addressed using the synthetic minority oversampling technique (SMOTE) applied to the training dataset. Model performance was evaluated using accuracy, sensitivity, specificity, and the area under the receiver operating characteristic curve (AUC).

**Results:**

Among 3274 neonates included in the study, the XGBoost model demonstrated the best predictive performance on the independent test set (n=655, 20%), achieving an accuracy of 94% (616/655 correct predictions, 95% CI 92% to 96%), sensitivity of 98% (95/97 sepsis cases correctly identified, 95% CI 96% to 99%), and an AUC of 0.98 (95% CI 0.97 to 0.99). Decision trees provided interpretable classification rules with moderate performance, whereas neural networks showed lower discriminative ability, with an AUC of 0.81 (95% CI 0.78 to 0.84). Important predictive features included C-reactive protein, platelet count, and gestational age.

**Conclusions:**

XGBoost demonstrated strong predictive performance in this retrospective cohort, supporting its potential as a foundation for future prospective clinical decision support tools. External validation and prospective studies are required before clinical implementation.

## Introduction

Neonatal sepsis (NS) remains a major contributor to neonatal mortality worldwide, causing approximately 550,000 deaths per year (15% of neonatal deaths) in low- and middle-income countries (LMICs) [1,2]. Preterm neonates and those with low birth weight are particularly vulnerable due to immature immune systems and prolonged exposure to invasive procedures [3]. Early prediction and detection of sepsis are critical for improving outcomes; however, challenges arise from nonspecific clinical presentations; delays in conventional diagnostics such as blood cultures; and the limited reliability of biomarkers, including C-reactive protein (CRP), procalcitonin, and complete blood count [3-7]. The increasing prevalence of multidrug-resistant organisms further complicates treatment, emphasizing the need for innovative tools to optimize antibiotic use and address antimicrobial resistance (AMR) [8].

Machine learning (ML) has emerged as a promising approach for early sepsis prediction by integrating clinical, laboratory, and electronic health record (EHR) data [9-13]. Algorithms such as Extreme Gradient Boosting (XGBoost) and neural networks can detect complex patterns in large datasets, enabling rapid risk assessment and potential early intervention [12-14]. Challenges such as class imbalance, where nonsepsis cases dominate datasets, can reduce model sensitivity, while overfitting may limit generalizability in single-center cohorts [15-17]. Techniques including the synthetic minority oversampling technique (SMOTE) and regularization address these issues but remain underexplored in LMIC contexts [4,18,19].

Previous studies have primarily focused on high-income countries [19-22], with limited evidence from regions such as Jordan, where resource constraints and unique epidemiological profiles necessitate context-specific approaches [3]. Emerging research in other LMICs has begun to address this gap, with ML models for NS showing promise in diverse settings, including India [23], Uganda [24], and Ethiopia [25]. In this context, this study aimed to evaluate ML models for predicting culture-confirmed NS in a Jordanian tertiary neonatal intensive care unit (NICU), addressing key diagnostic challenges in resource-limited settings. Specifically, we aimed to identify important predictors through feature importance analysis, assess model performance in the presence of class-imbalanced data, and explore strategies to enhance interpretability and generalizability in LMIC contexts. To support these objectives, we implemented an adaptable framework integrating SMOTE with XGBoost, providing a reproducible approach for early sepsis detection and future multicenter validation.

## Methods

### Ethical Considerations

The data used in this study were originally collected for a prior investigation on trends in NS organisms and antimicrobial sensitivity (Institutional Review Board of Jordan University [IRB] approval 10/2023/16360). For this secondary analysis, deidentified datasets were repurposed to predict NS using ML. All procedures adhered to international and national ethical standards, including the Declaration of Helsinki. Data were anonymized and accessible only to the research team. Because the study involved retrospective records without direct patient contact, a waiver of informed consent was approved by the Scientific Research Ethics Committee University of Jordan, School of Medicine (IRB 787/2023/76; renewal 4531/2025/67).

### Study Design and Population

**Figure 1.**
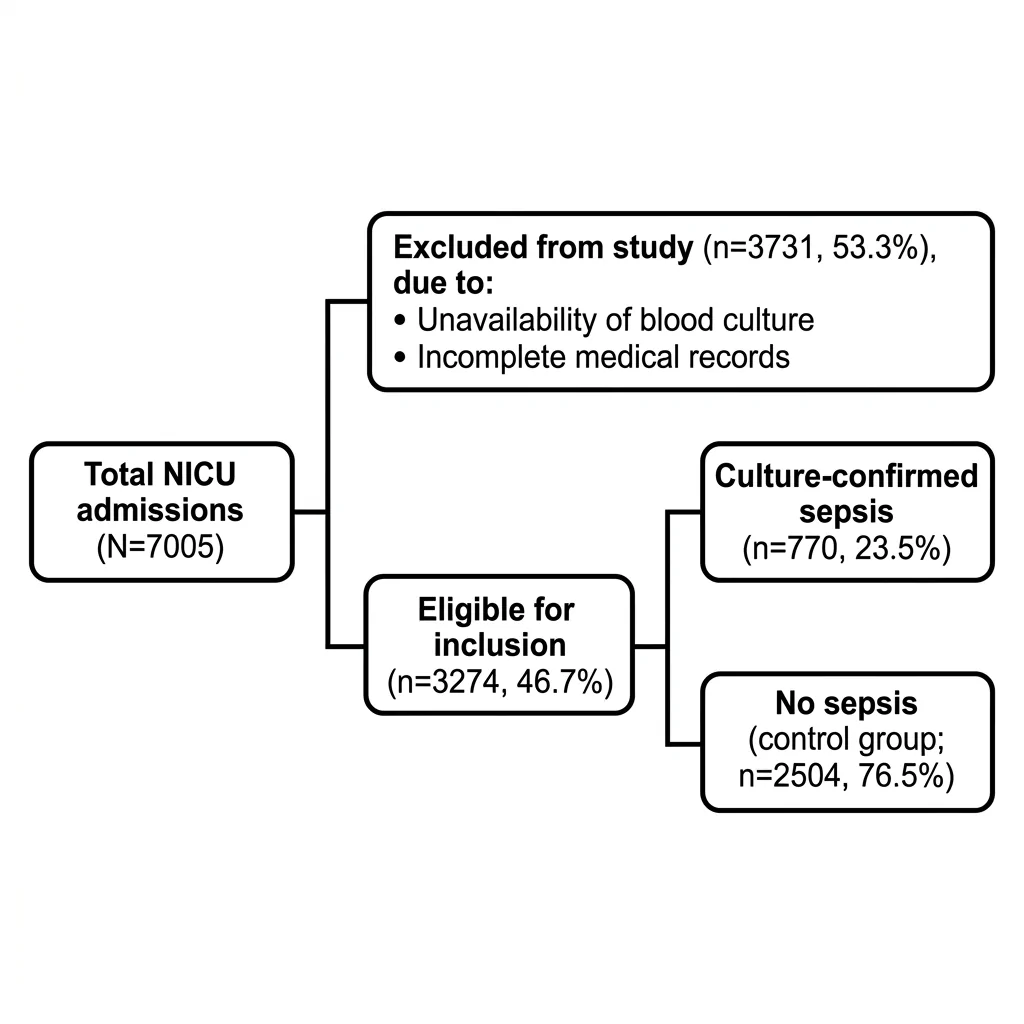
Flow diagram of participant selection. NICU: neonatal intensive care unit.

A retrospective cohort study was conducted at the 34-bed NICU of Jordan University Hospital, which admits approximately 1000 neonates annually. Between October 2018 and April 2024, 7005 neonates were admitted. Of these 7005 neonates, 3731 (53.3%) were excluded due to unavailability of blood culture data, leaving 3274 (46.7%) eligible for inclusion in the 3 predictive ML models for NS evaluation. Among the 3274 included cohort, 770 (23.5%) had culture-confirmed sepsis. Figure 1 shows the cohort flow diagram.

The eligibility criteria and definition of sepsis used to determine inclusion and classify the study population were as follows:

- Inclusion criteria: neonates aged <28 days at admission who had blood cultures collected and complete clinical, laboratory, and demographic data were included in the study.
- Exclusion criteria: neonates with incomplete records or no blood culture data were considered ineligible and therefore excluded from the study.
- Sepsis definition: sepsis status was classified based on the presence or absence of culture-confirmed sepsis, with sepsis=1 indicating culture-confirmed sepsis and sepsis=0 indicating no clinical or laboratory evidence of sepsis.

### Data Sources and Categories

Data were extracted from the NICU EHR system and included the following variables:

- Demographics: including birth weight, gestational age (GA), delivery mode, APGAR (appearance, pulse, grimace, activity, and respiration) scores at 1 and 5 minutes, sex, and exclusive breastfeeding
- Clinical indicators: including prolonged rupture of membranes (PROM), respiratory distress, and neonatal resuscitation
- Laboratory variables: including blood culture, CRP, white blood cell (WBC) count, absolute neutrophil count, neutrophil-to-lymphocyte ratio, platelet count (PLT), and hemoglobin
- Procedural data: including central venous catheter placement, invasive ventilation, surgery, blood transfusion, lumbar puncture, and chest tube insertion

A complete description of all predictor variables, including definitions, measurement units, timing, and transformations applied, is provided in Table S1 in Multimedia Appendix 1.

### Preprocessing Steps

The dataset of 3274 neonates was randomly divided into training and testing sets using stratified sampling to preserve the original sepsis class distribution (23.5% sepsis in both subsets), with a training set comprising 2619 (80%) neonates and a testing set comprising 655 (20%) neonates.

#### Handling Missing Data

Missing values were imputed using the mean for continuous variables and the mode for categorical variables to provide a consistent preprocessing framework across all evaluated ML models. This deterministic approach was selected to ensure reproducibility and comparability between algorithms. However, we acknowledge that missingness in routinely collected clinical data is frequently informative rather than completely random. Consequently, simple imputation may not fully capture the underlying data-generating mechanisms and may influence predictive performance. More sophisticated approaches, such as multiple imputation, model-based imputation, or missingness-aware ML algorithms, should be evaluated in future studies [9,17].

#### Normalization

Continuous variables were scaled using minimum-maximum normalization (0-1) to ensure a uniform feature range [9,17].

#### Categorical Encoding

Categorical variables (eg, mode of delivery and sex) were 1-hot encoded to enable integration with tree-based and neural network algorithms.

#### Temporal Alignment

To minimize temporal leakage, all predictor variables included in model development were restricted to information available at or before the time of blood culture sampling, which represented the prediction time point. For procedural variables (eg, central line insertion, intubation, and catheter-related procedures), the EHR contained temporal indicators (date [DD/MM/YYYY] and time [HH:MM]) specifying whether the procedure occurred before sample collection. Only procedures documented before blood culture sampling were eligible for inclusion in the predictive models. Variables occurring after the prediction time, such as exclusive breastfeeding at discharge, were excluded from model development and retained only for descriptive analyses.

### Demographic and Clinical Characteristics

#### Overview

Baseline demographic and clinical characteristics of the cohort are summarized in Table S2 in Multimedia Appendix 2. In the cohort of 3247 neonates, 1334 (40.7%) were female, and 1940 (59.3%) were male, with a mean postnatal age at sampling of 4.88 (SD 9.9) days and a mean GA of 35.53 (SD 3.4) weeks. Exclusive breastfeeding was reported in 1574 (48.1%) neonates, and PROMs (>18 hours) occurred in 443 (13.5%) cases.

#### Birth Weight Distribution

The distribution of 3274 neonates according to birth weight category was as follows:

- Extremely low birth weight (<1000 g): 109 (3.3%) neonates
- Very low birth weight (1000-1500 g): 229 (7%) neonates
- Low birth weight (1500-2500 g): 1084 (33.1%) neonates
- Normal birth weight (2500-4000 g): 1793 (54.8%) neonates
- High birth weight (>4000 g): 56 (1.7%) neonates

#### Delivery and Clinical Outcomes

The mean APGAR scores were 7.58 (SD 1.25) at 1 minute and 8.84 (SD 0.69) at 5 minutes. In the cohort of 3274 cases, delivery was by cesarean section in 2399 (73.3%) cases and by vaginal delivery in 875 (26.7%) cases. Clinical interventions included intubation in 296 (9%) neonates, umbilical venous catheter placement in 392 (12%) neonates, central line placement in 30 (0.9%) neonates, blood transfusion in 323 (9.9%) neonates, cardiopulmonary resuscitation in 135 (4.1%) neonates, and surgery in 179 (5.5%) neonates. Mortality occurred in 171 (5.2%) neonates.

### ML Models

#### Overview and Workflow

Three ML models—XGBoost (version 2.1.0; XGBoost Developers, 2024), neural networks, and decision trees—were applied to predict culture-confirmed NS. The modeling workflow included feature selection, class imbalance handling, and overfitting mitigation to ensure robust and reproducible predictions.

#### Feature Selection

XGBoost was used for recursive feature elimination and feature importance analysis to identify predictors critical to NS [17,19].

#### Addressing Class Imbalance

The dataset showed a strong imbalance between nonsepsis (majority) and sepsis (minority) cases. To mitigate this imbalance, the following approaches were used: oversampling, in which the minority class (sepsis=1) was oversampled using SMOTE, applied only to training folds during model training; and class weights, in which the loss functions in XGBoost and neural networks incorporated class weights to improve sensitivity to the minority class without biasing overall predictions [18,19].

#### Overfitting Mitigation

##### XGBoost

Overfitting in XGBoost was mitigated using L1 (reg_alpha) and L2 (reg_lambda) regularization, with the maximum tree depth limited to 5.

##### Neural Network

Overfitting in the neural network was mitigated using a multilayer perceptron (MLP) with 2 hidden layers of 100 neurons each, rectified linear unit (ReLU) activation, L2 regularization, early stopping, and feature scaling.

##### Decision Trees

Overfitting in the decision tree models was mitigated by limiting the maximum depth to 5, setting the minimum samples per split to 10, and setting the minimum number of samples per leaf to 5.

### XGBoost

XGBoost is an implementation of gradient-boosted decision trees that builds multiple trees sequentially, where each subsequent tree corrects the errors of the previous tree. The model incorporates L1 and L2 regularization to prevent overfitting. Refer to Multimedia Appendix 3 for the complete mathematical formulation, including the regularized objective function and regularization terms.

### Neural Network

**Figure 2.**
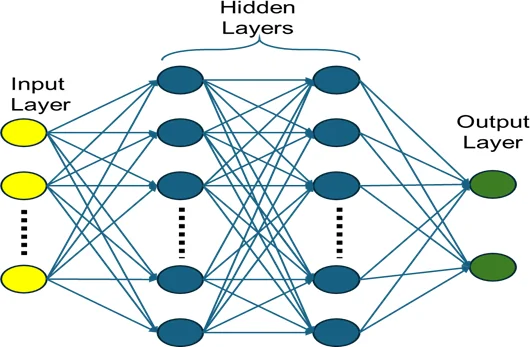
Two-hidden-layer multilayer perceptron architecture for neonatal sepsis, with rectified linear unit activation.

The neural network used in this study is an MLP classifier with an input layer, 2 hidden layers of 100 neurons each using ReLU activation, and an output layer for binary classification. The model was optimized using the Adam optimizer. Refer to Multimedia Appendix 3 for the mathematical formulation of neuron activation and loss functions. Figure 2 shows the neural network architecture.

### Decision Tree

The decision tree classifier splits data at each node based on criteria that maximize information gain or minimize Gini impurity. Tree depth was limited to 5, with minimum samples per split of 10 and minimum samples per leaf of 5 to prevent overfitting. Refer to Multimedia Appendix 3 for the mathematical formulations of Gini impurity and information gain.

### Evaluation of Model Performance

For XGBoost, 95% CIs for accuracy, recall (sensitivity), and specificity were calculated using the Wilson score method, while area under the receiver operating characteristic curve (AUC) CIs were estimated using the DeLong method. For the neural network and decision tree models, all performance metric CIs (accuracy, sensitivity, specificity, precision, recall, *F*_1_-score, and AUC) were estimated using bootstrap resampling with 1000 iterations.

## Results

### Feature Importance

The feature importance analysis was obtained from the XGBoost algorithm. Table 1 presents the contribution of each feature to predicting sepsis, ranked by their respective *F* scores. The *F* score indicates how many times a feature is used to perform a data split across all decision trees in the XGBoost model. Features with higher *F* scores are more frequently chosen for partitioning, exerting a greater impact on the model’s predictions, while features with lower *F* scores have less influence.

**Table 1.** Feature importance and their *F* scores derived from the Extreme Gradient Boosting (XGBoost) model for neonatal sepsis prediction.

Features	*F* scores
C-reactive protein (mg/L)	826.0
Platelets count (×10⁹/L)	641.0
Hemoglobin (g/dL)	561.0
White blood cell count (×10⁹/L)	497.0
Neutrophils (%)	416.0
Gestational age (wks)	395.0
Absolute neutrophils count (×10⁹/L)	391.0
Lymphocyte (%)	387.0
Red blood cell count (×10¹²/L)	347.0
Neutrophil-to-lymphocyte ratio	250.0
APGAR^a^ at 1 minute	196.0
Duration of umbilical venous catheterization (days)	151.0
Age at intubation (days)	141.0
Mode of delivery	82.0
Exclusive breastfeeding at discharge^b^	78.0
Patient age at admission (days)	69.0
Umbilical line age (days)	56.0
Sex	52.0
APGAR at 5 minutes	43.0
Age at central line insertion (days)	36.0
Low birth weight	33.0
Very low birth weight	28.0
Surgical procedure	25.0
Extremely low birth weight	22.0
Normal birth weight	22.0
Prolonged rupture of membranes	21.0
Umbilical venous catheter	18.0
Intervention	16.0
Intubation in relation to blood culture sampling	12.0
Chest tube	9.0
Hepatitis B vaccine	1.0
Central line insertion in relation to blood culture sampling	1.0
Blood transfusions	0.0

a APGAR: appearance, pulse, grimace, activity, and respiration.

b Exclusive breastfeeding at discharge was included in model-derived feature importance only. It is not a clinically valid predictor for real-time sepsis prediction because it was not temporally available. No causal or clinical inference should be made from this variable.

The most significant predictors of sepsis included CRP (*F* score=826.0), PLT (*F* score=641.0), hemoglobin (*F* score=561.0), and GA (*F* score=395.0). Moderately influential predictors were WBC count (*F* score=497.0) and neutrophil percentage (*F* score=416.0), with elevated neutrophils being strongly associated with bacterial invasion, consistent with the pathophysiology of sepsis. The APGAR score at 1 minute (*F* score=196.0) correlated with perinatal stress, such as hypoxia or trauma, highlighting birth complications as a potentially modifiable risk factor.

Exclusive breastfeeding at discharge (*F* score=78.0) appeared in the feature importance analysis derived from the trained XGBoost model; however, it is not temporally available at the time of prediction and therefore is not a clinically valid predictor for real-time sepsis detection.

In contrast, central line insertion and chest tube interventions had much lower *F* scores, suggesting a smaller role in model decisions. This analysis highlights which clinical parameters are most vital for predicting NS.

### Overall Model Performance

All 3 ML models were evaluated for their ability to predict NS. XGBoost showed the best accuracy and discriminatory power, followed by the decision tree and neural network models. Across models, the incorporation of class imbalance handling, feature selection, and overfitting mitigation contributed to improved sensitivity for minority sepsis cases while maintaining balanced performance for the majority class. The overall trends suggest that tree-based models, particularly gradient boosting, may be better suited for structured neonatal EHR data, while neural networks demonstrated moderate performance likely limited by dataset size and hyperparameter sensitivity.

The results are summarized in Table 2. Each model offered unique strengths and trade-offs regarding accuracy, recall, and precision, reflecting its suitability for this specific task.

**Table 2.** Comparative performance metrics of machine learning models for neonatal sepsis prediction^a^.

Models	Accuracy, % (95% CI)	Precision	Recall (95% CI)	*F*_1_-score	AUC^b^ (95% CI)
Extreme Gradient Boosting	94 (92-96)	0.91	0.98 (0.96-0.99)	0.94	0.98 (0.97-0.99)
Neural network	75 (71-78)	0.74	0.73 (0.69-0.76)	0.74	0.81 (0.78-0.84)
Decision tree	80 (77-83)	0.77	0.84 (0.81-0.87)	0.80	0.86 (0.83-0.89)

a All metrics were calculated on the independent test set (N=655).

b AUC: area under the receiver operating characteristic curve.

### XGBoost Performance

XGBoost demonstrated superior performance among the 3 models. It achieved a mean cross-validation accuracy of 93.7% (SD 1.2%) and a test accuracy of 94% (95% CI 92%-96%; 616/655, 94% correct predictions), making it the most effective model for this task. For sepsis=0, precision was 0.98 (95% CI 0.96-0.99) and recall was 0.91 (95% CI 0.88-0.93), while for sepsis=1, precision was 0.91 (95% CI 0.88-0.93) and recall was 0.98 (95/97, 98% sepsis cases, 95% CI 0.96 to –0.99). The *F*_1_-score for both classes was 0.94, reflecting a strong balance between precision and recall.

**Figure 3.**
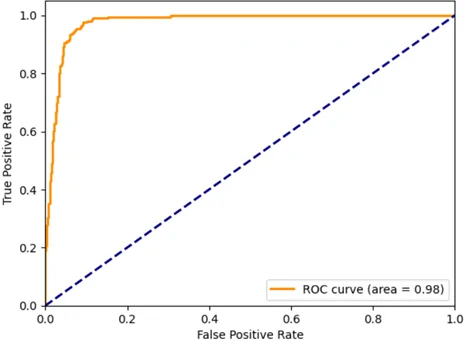
Receiver operating characteristic (ROC) curve for the Extreme Gradient Boosting (XGBoost) model demonstrating high discriminatory power.

Figure 3 shows the receiver operating characteristic (ROC) curve for XGBoost, with an AUC of 0.98 (95% CI 0.97-0.99), indicating excellent discriminatory power. The curve rises steeply near the y-axis, demonstrating the model’s ability to correctly classify positive cases while maintaining a minimal false-positive rate. Its proximity to the upper-left corner emphasizes high sensitivity and specificity. These results underscore XGBoost’s robustness in handling structured and imbalanced datasets. Its incorporation of class weights and regularization enables a focus on minority classes without compromising overall accuracy, and minimal variation in cross-validation scores confirms stability and generalization capability.

### Neural Network Performance

**Figure 4.**
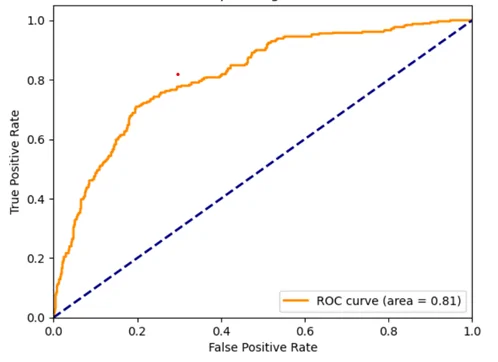
Receiver operating characteristic (ROC) curve for the neural network model in neonatal sepsis prediction.

The neural network model, despite its flexibility in modeling complex relationships, underperformed compared to XGBoost. It achieved a test accuracy of 75% (95% CI 71%-78%; 491/655, 75% correct predictions). For sepsis=0, precision was 0.76 (95% CI 0.73-0.79) and recall was 0.77 (95% CI 0.74-0.80). For sepsis=1, precision was 0.74 (95% CI 0.71-0.77) and recall was 0.73 (71/97, 73% sepsis cases, 95% CI 0.70 to –0.76). Figure 4 shows the ROC curve for the neural network, with an AUC of 0.81 (95% CI 0.78-0.84). The curve demonstrates a good, although not perfect, ability to discriminate between positive and negative cases. Its upward trajectory reflects the model’s capacity to achieve a high true-positive rate while maintaining a moderate false-positive rate, although deviations from the upper-left corner indicate some misclassification. The lower performance likely stems from the limited dataset size, the neural network’s sensitivity to hyperparameters, and the structured nature of the data, which tree-based models such as XGBoost handle more efficiently. Despite these limitations, the neural network demonstrated reasonable predictive capability and could potentially improve with hyperparameter tuning, feature scaling, or a larger dataset.

### Decision Tree Performance

Decision trees are inherently interpretable, making them valuable in clinical applications where understanding model decisions is critical. Pruning techniques, including limiting tree depth and controlling the minimum number of samples per split and leaf, mitigated overfitting and ensured generalization.

**Figure 5.**
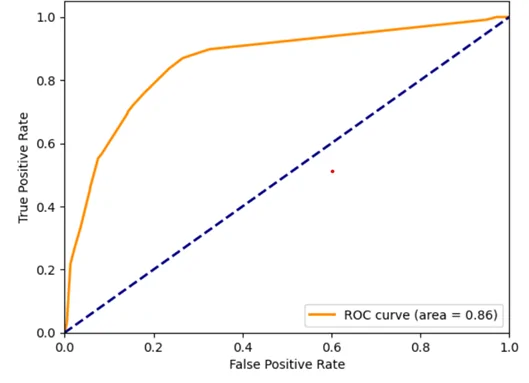
Receiver operating characteristic (ROC) curve for the decision tree model in neonatal sepsis classification.

The decision tree achieved a mean cross-validation accuracy of 79.1% and a test accuracy of 80% (95% CI 77%-83%; 524/655, 80% correct predictions). Precision and recall were relatively balanced across both classes. For sepsis=0, precision was 0.83 (95% CI 0.80-0.86) and recall was 0.77 (95% CI 0.74-0.80), while for sepsis=1, precision was 0.77 (95% CI 0.74-0.80) and recall was 0.84 (81/97, 84% sepsis cases, 95% CI 0.81 to –0.87). The *F*_1_-score was 0.80 for both classes. Figure 5 shows the ROC curve, with an AUC of 0.86 (95% CI 0.83-0.89), reflecting good discriminative performance. The curve demonstrates the model’s ability to correctly identify positive cases, although a moderate false-positive rate is observed. Although its performance was lower than that of XGBoost, the decision tree provides an interpretable and clinically useful alternative for NS prediction.

## Discussion

In this retrospective cohort study of 3274 neonates in a Jordanian tertiary NICU, the XGBoost ML model achieved excellent discrimination for culture-confirmed NS, with an accuracy of 94% (616/655 correct predictions, 95% CI 92%-96%), a sensitivity of 98% (95/97 sepsis cases, 95% CI 96%-99%), a specificity of 91% (508/558 nonsepsis cases, 95% CI 89%-93%), and an AUC of 0.98 (95% CI 0.97-0.99), outperforming neural networks (AUC 0.81, 95% CI 0.78-0.84) and decision trees (AUC 0.86, 95% CI 0.83-0.89).

### Model Performance and Clinical Implications

This study highlights the transformative potential of ML for NS prediction, especially in resource-constrained settings. XGBoost emerged as the top-performing model, achieving consistent performance across all folds with a mean accuracy of 93.7% (SD 1.2%), a mean AUC of 0.98 (SD 0.01), and a mean sensitivity of 0.97 (SD 0.02). Minimal fold-wise variance confirmed strong generalizability, with better performance than neural networks (AUC 0.81, 95% CI 0.78-0.84) and decision trees (AUC 0.86, 95% CI 0.83-0.89).

These findings align with prior studies identifying gradient-boosting algorithms as particularly well-suited for structured medical data due to their robustness against class imbalance and capacity for regularization [19,22]. A recent systematic review also identified ensemble methods such as XGBoost among the top-performing models, despite heterogeneity in study designs and outcomes [26]. Our model (AUC 0.98) compares favorably with LMIC-based studies, including a regression nomogram from Ethiopia (AUC 0.81) [25] and an electronic medical record–based model from Uganda with moderate performance [24]. Together, these findings suggest that gradient boosting may outperform traditional regression approaches in LMIC settings, although external validation remains necessary.

Feature importance analysis further reinforced clinical relevance, with CRP, PLT, and GA identified as top predictors, consistent with established biomarkers for early sepsis detection [3,18].

Decision trees, while slightly less accurate (accuracy 80%, 95% CI 77%-83%; AUC 0.86, 95% CI 0.83-0.89), provided interpretable decision pathways that may support clinical decision-making and bridge the gap between algorithmic outputs and bedside practice [22].

### Addressing Methodological Challenges

Class imbalance, a pervasive issue in medical ML, was mitigated using SMOTE oversampling and class-weighted loss functions, improving sensitivity to sepsis cases (recall 0.98, 95% CI 0.96‐0.99) without compromising specificity [17,18]. Overfitting risks were addressed via L1/L2 regularization in XGBoost and neural networks, alongside constrained tree depth in decision trees [17,19]. Cross-validation ensured internal generalizability, although the single-center, retrospective design limits external validity. Collectively, these strategies may enhance model reliability and adhere to best practices for ML implementation in LMICs [15,20-26].

### Strengths

This study demonstrates high diagnostic accuracy, with XGBoost achieving an AUC of 0.98 (95% CI 0.97-0.99), surpassing conventional biomarker-based approaches and enabling timely identification of NS. The model effectively identifies actionable predictors, including CRP, PLT, and GA, which align with clinical workflows and support targeted monitoring. Additionally, the study effectively addressed class imbalance using SMOTE and class-weighted loss functions, improving detection of sepsis cases without compromising performance for the majority class. Collectively, these methodological innovations may enhance the robustness and clinical relevance of the predictive framework for LMIC neonatal populations.

### Limitations

The retrospective, single-center design may introduce selection bias and limits generalizability to other NICU settings or populations. The analysis was restricted to culture-positive cases, which may exclude culture-negative sepsis, limiting the ability to predict sepsis in its earliest stages. The timing of feature availability relative to sepsis onset is also uncertain, which may impact true early prediction. Although temporal constraints were applied to all procedural variables included in the predictive models, some laboratory and intervention-related variables may still partially reflect clinician-driven diagnostic or treatment decisions rather than purely biological manifestations of NS. Consequently, feature importance should be interpreted as reflecting predictive utility within the study cohort rather than causal relationships. Although cross-validation reduces overfitting, external validation in multicenter prospective cohorts is necessary to confirm model performance. Exclusive breastfeeding at discharge was included only in descriptive analyses and feature importance. It was not available at the time of prediction and is not a clinically valid predictor for real-time sepsis detection due to temporal misalignment. An additional limitation relates to missing data handling. Although mean and mode imputation provided a simple and consistent preprocessing strategy across all ML models, this approach assumes that missing values can be adequately represented by measures of central tendency. Because missingness in EHRs is often informative and not completely at random, the present results should be interpreted with this limitation in mind. Future studies should investigate advanced imputation techniques, such as multiple imputation, missing-indicator approaches, sensitivity analyses under different missingness assumptions, and missingness-aware learning methods, to evaluate their impact on predictive performance and model robustness. Finally, algorithmic transparency and data privacy considerations could influence clinical trust and adoption, highlighting the need for interpretability tools and secure integration into EHR systems.

### Future Directions

Prospective multicenter validation is essential to ensure the robustness and generalizability of the models across diverse geographic and demographic neonatal cohorts. Recent comprehensive reviews have highlighted both the potential and the challenges of implementing AI for NS in resource-limited settings, emphasizing the need for context-specific solutions and robust external validation [27]. Integrating AMR data into ML frameworks could optimize antibiotic stewardship and support clinical decision-making in LMIC settings. Region-specific model tuning, using localized datasets and tailored algorithms, could enhance predictive accuracy and applicability for neonatal populations with unique epidemiological characteristics. Furthermore, the adoption of interpretability tools such as Shapley Additive Explanations (SHAP) or Local Interpretable Model-Agnostic Explanations (LIME) can improve clinician trust, facilitate integration into EHR systems, and support transparent, actionable insights for bedside decision-making. These interpretability considerations align with recommendations from recent scoping reviews, which emphasize that clinician trust and model transparency are critical barriers to real-world implementation of ML for NS [28].

### Conclusions

This study represents the first ML-driven approach tailored to NS prediction in Jordan, addressing diagnostic gaps and highlighting the need for region-specific models in LMICs. Our findings demonstrate that ML models, particularly XGBoost, can integrate heterogeneous clinical, laboratory, and demographic data to provide actionable predictions, potentially enabling earlier interventions and improved neonatal outcomes. Although challenges such as class imbalance, data limitations, and ethical considerations remain, methodological innovations in regularization, oversampling, and interpretability may enhance model reliability and usability. Future work should focus on prospective multicenter validation, incorporation of AMR patterns, and equitable model deployment to support clinical decision-making and reduce neonatal mortality in resource-limited settings.

## Supplementary material

10.2196/88732 Multimedia Appendix 1 Description of predictor variables and definitions.

10.2196/88732 Multimedia Appendix 2 Baseline characteristics.

10.2196/88732 Multimedia Appendix 3 Mathematical formulations of Extreme Gradient Boosting, neural network, and decision tree models.

## Abbreviations

AMR: antimicrobial resistance
APGAR: appearance, pulse, grimace, activity, and respiration
AUC: area under the receiver operating characteristic curve
CRP: C-reactive protein
EHR: electronic health record
GA: gestational age
IRB: institutional review board
LIME: Local Interpretable Model-Agnostic Explanations
LMIC: low- and middle-income country
ML: machine learning
MLP: multilayer perceptron
NICU: neonatal intensive care unit
NS: neonatal sepsis
PLT: platelet count
PROM: prolonged rupture of membranes
ReLU: rectified linear unit
ROC: receiver operating characteristic
SHAP: Shapley Additive Explanations
SMOTE: synthetic minority oversampling technique
WBC: white blood cell
XGBoost: Extreme Gradient Boosting
