# Supplementary material for: Machine Learning–Based Prediction of Culture-Confirmed Neonatal Sepsis in a Tertiary Neonatal Intensive Care Unit: Retrospective Cohort Study
Source: JMIR Med Inform. 2026 Sep 14;14:e88732. doi: 10.2196/88732 (PMC13573726; doi:10.2196/88732)
Supplement: Supplementary file 1 [file medinform-v14-e88732-s001.docx]

**S1 Table. Description of predictor variables and definitions.**

| **Variable Name** | **Type** | **Definition** | **Unit of Measurement** | **Timing of Measurement** | **Method / Instrument Used** | **Transformation Applied** |
| --- | --- | --- | --- | --- | --- | --- |
| **Serial no** | Identifier* | Unique serial number for the record | N/A | N/A | EHR** | None |
| **Patient id** | Identifier* | Unique hospital Identifier* | N/A | N/A | EHR** | None |
| **Admission no.** | Identifier* | Hospital admission number | N/A | N/A | EHR** | None |
| **Patient name** | Identifier* | Name of patient (not used in analysis) | N/A | N/A | EHR** | None |
| **Birth date** | Identifier* | Date of birth | Date format | At birth | EHR** | None |
| **Birth date-time** | Identifier* | Exact birth date & time | Date/Time | At birth | EHR** | None |
| **Gender** | Predictor | Biological sex | Categorical (male/female) | At birth | EHR** | Dichotomized |
| **GA (gestational age)** | Predictor | Gestational age | Weeks | At birth | EHR** | Used as continuous |
| **Birth weight** | Predictor | Weight at birth | Grams | At birth | EHR** | Categorized (ELBW, VLBW, LBW, NBW, HBW) |
| **Mode of delivery** | Predictor | Mode of delivery (NVD or CS) | Categorical | At birth | EHR** | Dichotomized |
| **Shift** | Identifier* | NICU nursing shift | Categorical (Shift A/B/C) | At birth | EHR** | None |
| **Admission date** | Identifier* | Date of hospital admission | Date format | At admission | EHR** | None |
| **Discharge date** | Identifier* | Date of discharge | Date format | At discharge | EHR** | None |
| **Length of stay** | Predictor | Duration of hospital admission | Days | Entire admission | EHR** | Calculated (Discharge – Admission) |
| **Request date** | Identifier* | Date when lab/clinical sepsis evaluation was requested | Date format | At clinical suspicion | EHR** | None |
| **Section** | Identifier* | NICU section/ward | Categorical | At admission | EHR** | None |
| **TERN*** | Identifier | Transport/Referral status | Categorical | At admission | EHR** | None |
| **APGAR at 1 min** | Predictor | Apgar score at 1 min | Score (0-10) | 1 min after birth | EHR** | Used as continuous |
| **APGAR at 5 min** | Predictor | Apgar score at 5 min | Score (0-10) | 5 min after birth | EHR** | Used as continuous |
| **UL ON / UL OFF** | Predictor | Date of UVC insertion and removal | Date format | During admission | EHR** | Used to calculate UVC duration |
| **CL ON / CL OFF** | Predictor | Date of central line insertion and removal | Date format | During admission | EHR** | Used to calculate central line duration |
| **INTUBATION** | Predictor | Need for intubation for mechanical ventilation | Categorical (Yes/No) | During admission | EHR** | Dichotomized |
| **Duration of UVC** | Predictor | Duration a UVC was in place | Number of days | During admission | EHR** | Calculated (UL OFF – UL ON) |
| **Age at Intubation** | Predictor | Patient's age when first intubated | Number of days | During admission | EHR** | Calculated (birth to intubation) |
| **Exclusive Breast Milk at Discharge** | Predictor | Whether patient was on exclusive breast milk at discharge | Categorical (Yes/No) | At discharge | EHR** | Dichotomized |
| **Patient Age at Admission** | Identifier | Patient's age at NICU admission | Number of days | At admission | EHR** | Calculated (birth to admission) |
| **UL Age** | Predictor | Patient's age when UVC was inserted | Days | During admission | EHR** | Calculated (birth to UL ON) |
| **Age at Central Line Insertion** | Predictor | Patient's age when central line inserted | Days | During admission | EHR** | Calculated (birth to CL ON) |
| **Surgical procedure** | Predictor | Whether a surgical procedure was performed | Categorical (Yes/No) | During admission | EHR** | Dichotomized |
| **Blood Transfusions** | Predictor | Whether a blood transfusion was administered | Categorical (Yes/No) | During admission | EHR** | Dichotomized |
| **UV (Umbilical Venous Catheter)** | Predictor | Presence of a UVC | Categorical (Yes/No) | During admission | EHR** | Dichotomized |
| **INT (Intervention)** | Predictor | One or more of: chest tube, abdominal paracentesis, or surgery | Categorical (Yes/No) | During admission | EHR** | Dichotomized |
| **Intubation in Relation to Culture** | Predictor | Intubation status of the neonate at the time of blood culture collection | Categorical (Yes/No) | During admission | EHR** | Dichotomized |
| **Chest Tube** | Predictor | Presence of a chest tube | Categorical (Yes/No) | During admission | EHR** | Dichotomized |
| **HBV (Hepatitis B Vaccine)** | Predictor | Administration of Hepatitis B vaccine | Categorical (Yes/No) | During admission | EHR** | Dichotomized |
| **Central Line in Relation to Culture** | Predictor | Central line status of the neonate at the time of blood culture collection | Categorical (Yes/No) | During admission | EHR** | Dichotomized |
| **Final result** | Outcome | Laboratory results of blood cultures | - | During admission | Laboratory (Culture) | None |
| **CRP** | Predictor | C-Reactive Protein level | mg/L | During admission | Laboratory (Immunoassay) | Used as continuous |
| **PL (Platelets)** | Predictor | Platelet count | 10^9/L | During admission | Laboratory | Used as continuous |
| **HB (Hemoglobin)** | Predictor | Hemoglobin concentration | g/dL | During admission | Laboratory | Used as continuous |
| **WBC** | Predictor | White Blood Cell count | 10^9/L | During admission | Laboratory | Used as continuous |
| **Neutrophils %** | Predictor | Percentage of neutrophils | % | During admission | Laboratory | Used as continuous |
| **Absolute Neutrophils Count** | Predictor | Absolute count of neutrophils | 10^9/L | During admission | Derived | Calculated (WBC * Neutrophils % / 100) |
| **Lymphocyte %** | Predictor | Percentage of lymphocytes | % | During admission | Laboratory | Used as continuous |
| **RBC** | Predictor | Red Blood Cell count | 10^12/L | During admission | Laboratory | Used as continuous |
| **NR (Neutrophil to Lymphocyte Ratio)** | Predictor | Ratio of neutrophils to lymphocytes | Unitless | During admission | Derived | Calculated (Neutrophils % / Lymphocyte %) |

*Identifiers were not used in analysis

**EHR: Electronic Health Records

N/A Not Applicable
