# Supplementary material for: Machine Learning–Based Prediction of Culture-Confirmed Neonatal Sepsis in a Tertiary Neonatal Intensive Care Unit: Retrospective Cohort Study
Source: JMIR Med Inform. 2026 Sep 14;14:e88732. doi: 10.2196/88732 (PMC13573726; doi:10.2196/88732)
Supplement: Supplementary file 2 [file medinform-v14-e88732-s002.docx]

# S2 Table. Baseline Characteristics

| **Characteristic** | **Distribution n(%)** |
| --- | --- |
| **Sex** | Females: 1,334 (40.7%)  Males: 1,940 (59.3%) |
| **Postnatal age at sampling (days)** | 4.88 ± 9.9 |
| **Gestational age (weeks)** | 35.53 ± 3.4 |
| **Exclusive breastfeeding** | 1,574 (48.1%) |
| **PROM >18 hours** | 443 (13.5%) |
| **Birth weight distribution** | |
| – ELBW (<1,000 g) | 109 (3.3%) |
| – VLBW (1,000–1,500 g) | 229 (7.0%) |
| – LBW (1,500–2,500 g) | 1,084 (33.1%) |
| – NBW (≥2,500 g) | 1,793 (54.8%) |
| – HBW (>4,000 g) | 56 (1.7%) |
| **APGAR score (mean ± SD)** | 1 min: 7.58 ± 1.25 5 min: 8.84 ± 0.69 |
| **Mode of delivery** | Cesarean: 2,399 (73.3%)  Vaginal: 875 (26.7%) |
| **Clinical interventions** | |
| – Intubation | 296 (9.0%) |
| – UVC insertion | 392 (12.0%) |
| – Central line | 30 (0.9%) |
| – Blood transfusion | 323 (9.9%) |
| – CPR | 135 (4.1%) |
| – Surgery | 179 (5.5%) |
| **Mortality** | 171 (5.2%) |
