# Supplementary material for: Machine Learning–Based Prediction of Culture-Confirmed Neonatal Sepsis in a Tertiary Neonatal Intensive Care Unit: Retrospective Cohort Study
Source: JMIR Med Inform. 2026 Sep 14;14:e88732. doi: 10.2196/88732 (PMC13573726; doi:10.2196/88732)
Supplement: Supplementary file 3 [file medinform-v14-e88732-s003.docx]

**MULTIMEDIA APPENDIX 1: Mathematical Formulations of Machine Learning Models**

**XGBoost**

XGBoost is an implementation of gradient boosted decision trees designed for high performance. In XGBoost, multiple decision trees are built sequentially, where each subsequent tree tries to correct the errors of the previous one. The model is trained by minimizing the following regularized objective function:

Where is the loss function for each prediction, and Ω(f_k_) is the regularization term for tree k, typically defined as:

Where T is the number of leaves in the tree, γ and λ are regularization parameters, and w represents the leaf weights.

**Neural Network**

Neural Networks are computational models inspired by the human brain, consisting of layers of interconnected nodes (neurons). A typical feedforward neural network has an input layer, one or more hidden layers, and an output layer.

The output of a neuron in a layer is computed as:

where: (w_i_) is the weight vector associated with the (i) − th	neuron, (x)	is the input vector, and (b_i_) is the bias term. The activation function (f(z_i_)) is then applied to the weighted sum (z_i_) to introduce non-linearity. A common choice of activation function is the Rectified Linear Unit (ReLU):

f(z_i_) = max (0, z_i_)

The neural network used in this paper is a Multi-Layer Perceptron (MLP) classifier designed for multi-class classification, consisting of an input, two hidden layers with 100 neurons each using ReLU activation, and an output layer for 2 classes. The model is optimized using the Adam optimizer.

**Decision Tree**

Decision Tree Classifier is a tree-like model where each internal node represents a test on an attribute, each branch represents the outcome of the test, and each leaf node represents a class label. The tree construction is guided by a criterion such as Gini impurity or Information Gain, calculated as:

Where (p_i_) is the proportion of samples belonging to class (i)	in the node.

where (H(S)) is the entropy of the dataset (S) and (S_i_) represents the subsets after the split.

The tree continues to split the data until it meets a stopping criterion, such as a maximum depth or a minimum number of samples per leaf.
